# Alcohol Disinhibition of Behaviors in *C. elegans*

**DOI:** 10.1371/journal.pone.0092965

**Published:** 2014-03-28

**Authors:** Stephen M. Topper, Sara C. Aguilar, Viktoria Y. Topper, Erin Elbel, Jonathan T. Pierce-Shimomura

**Affiliations:** Waggoner Center for Alcohol and Addiction Research, Cell & Molecular Biology, Department of Neuroscience, The University of Texas at Austin, Austin, Texas, United States of America; Virginia Commonwealth University, United States of America

## Abstract

Alcohol has a wide variety of effects on physiology and behavior. One of the most well-recognized behavioral effects is disinhibition, where behaviors that are normally suppressed are displayed following intoxication. A large body of evidence has shown that alcohol-induced disinhibition in humans affects attention, verbal, sexual, and locomotor behaviors. Similar behavioral disinhibition is also seen in many animal models of ethanol response, from invertebrates to mammals and primates. Here we describe several examples of disinhibition in the nematode *C. elegans*. The nematode displays distinct behavioral states associated with locomotion (crawling on land and swimming in water) that are mediated by dopamine. On land, animals crawl and feed freely, but these behaviors are inhibited in water. We found that additional behaviors, including a variety of escape responses are also inhibited in water. Whereas alcohol non-specifically impaired locomotion, feeding, and escape responses in worms on land, alcohol specifically disinhibited these behaviors in worms immersed in water. Loss of dopamine signaling relieved disinhibition of feeding behavior, while loss of the D1-like dopamine receptor DOP-4 impaired the ethanol-induced disinhibition of crawling. The powerful genetics and simple nervous system of *C. elegans* may help uncover conserved molecular mechanisms that underlie alcohol-induced disinhibition of behaviors in higher animals.

## Introduction

Ethanol (EtOH) is the most commonly abused drug, in part because of its culturally condoned role in disinhibiting behaviors that are suppressed during states of anxiety. This disinhibiting effect of EtOH results in a euphoric feeling of release, further reinforcing EtOH drinking habits. A variety of behaviors are disinhibited with EtOH consumption. For example, it is known to reduce anxiety [1], [2]. Previous work has found that acute EtOH intoxication decreases motor latency in simple “go/no go” trials [3], [4], [5]. EtOH also disinhibits behaviors critical for social interaction. Studies have shown that intoxication increases verbal expression and social bonding [6], [7]. There is also a wealth of research on the interaction between EtOH and sexual behaviors, with intoxicated individuals reporting higher sexual arousal and an increase in risky sexual behaviors [8], [9]. Disinhibition is a common, sometimes desired, effect of EtOH consumption in humans.

While the phenomenon of disinhibition by EtOH in humans has been known for some time, studying the neural mechanisms underlying these behaviors relied upon the development of appropriate animal models. To this end, researchers have established a variety of animal models that display disinhibition in response to EtOH. In rodent models, EtOH disinhibits locomotor patterns, often measured through the transient increase in total movement during acute intoxication as well as grooming [10]–[12]. Stress has also been shown to potentiate disinhibiting effects of EtOH, with stressed animals displaying an increase in EtOH-induced locomotion [12]. Several rodent studies reported relief of stress-induced behavioral inhibition via EtOH. In mice and rats, EtOH relieves stress-induced inhibition of a number of behaviors. Animals exposed to isolation stress displayed anxiety behaviors, assessed as reduced entries and time spent in the open arm in an elevated plus-maze test, which were partially relived by EtOH intoxication [13], [14]. Exposure to EtOH also relieves the impairment of social investigation, social preference, and spatial memory, induced by chronic restraint stress [12], [15]. Animals bred to prefer EtOH show a high baseline level of anxiety in the elevated plus-maze test, which is reversed by EtOH administration [16], [17]. In addition to mammalian models, evidence of EtOH-induced disinhibition has also been noted in the invertebrate model Drosophila. EtOH was shown to disinhibit sexual and locomotor behaviors in flies [18]. In this study, it was shown that repeated EtOH exposure disinhibited male-male courtship, a behavior unseen in normal flies. Thus, disinhibition is a common feature of EtOH intoxication across many different species.

Efforts to uncover the neuromolecular basis of EtOH-induced disinhibition have focused on the dopaminergic pathway. The dopaminergic system has been shown to be a key component of EtOH-induced disinhibition in mammalian and invertebrate models. Two decades ago, EtOH intoxication was shown to increase dopamine levels, measured via microdialysis, in the nucleus accumbens [19], [20]. It was later shown that this increase was due to excess dopamine release from the ventral tegmental area [21], [22]. Microinjection of dopamine receptor antagonists, including those that target D1 dopamine receptors, into the nucleus accumbens reduced responses to EtOH-paired stimuli, suggesting a role for these receptors in reward [23]–[25]. Dopamine release in the nucleus accumbens is also associated with locomotor disinhibition [26]. Pretreatment with dopamine reuptake inhibitors or D1 receptor agonists has been shown to sensitize animals to locomotor disinhibition, though this has not been consistently shown [27]–[29]. A recent study in flies, however, showed a similar role for D1 receptors in locomotor disinhibition [30]. Likewise, dopamine signaling was also shown to be involved in EtOH-induced disinhibition of male-male courtship in Drosophila [19].

In the present study, we examined whether EtOH induces disinhibition in the model nematode *Caenorhabditis elegans* and if the dopaminergic system was similarly implicated in these effects. Many studies have demonstrated the utility of the nematode *C. elegans* as a simple model to examine conserved molecular bases for behavioral responses to EtOH. While *C. elegans* cannot effectively model the full complexities of alcohol addiction in humans, the nematode has been used to model important aspects of EtOH abuse. During acute intoxication, worms exposed to EtOH display a gradual, dose-dependent decline in locomotor activity, similar to the depressive effects of EtOH seen in other animals [31], [32]. Importantly, the internal dose of EtOH that elicits this behavioral change is equivalent to that in humans as well as in rodent models of intoxication, suggesting that the underlying molecular targets may be the same. *C. elegans* also displays acute tolerance to EtOH, as evidenced by a recovery of locomotor behaviors after 30 minutes of intoxication [33]. Withdrawal from EtOH alters a number of behaviors. An increase in a social behavior, apparent as animals clumping together, has been observed during withdrawal [34]. Mitchell *et al.*, (2010) catalogued a number of locomotor defects upon withdrawal, including altered posture and an impaired ability to navigate towards food [34]. Thus, *C. elegans* has been shown to display many aspects of EtOH responses.

For this study, we chose a liquid immersion assay because *C. elegans* displays distinct subsets of behaviors on land, which are controlled by dopamine and are inhibited in aquatic environments [35], [36]. On land, the worm displays the crawling locomotor gait that is characterized by tight, low frequency bends, as well as a number of associated feeding behaviors. In water, the worm switches to a distinct swimming gait characterized by shallow and high frequency bends, and cessation of crawl-associated feeding behaviors [35], [36]. Initiation of crawling is dependent on the D1-like dopamine receptors DOP-1 and DOP-4, as evident by cessation of forward movement following immersion from water in mutant animals that lack these receptors [35]. Likewise, crawl-associated behaviors can be induced during immersion in water in wild-type animals by external application of dopamine or photostimulation of dopamine neurons with optogenetics [35], [36]. In the present study, we found that additional crawl-associated behaviors are also inhibited during immersion in water. Application of EtOH to worms in water resulted in disinhibition of crawling and associated behaviors. Disinhibition of several of these behaviors was reliant on dopamine signaling.

## Results

### Immersion in liquid inhibits a subset of behaviors in *C. elegans*

Before investigating the potential effects of EtOH on disinhibiting behaviors in *C. elegans*, we quantified a collection of behaviors that the worm displays on semi-moist agar plates (hereafter called the “on land” condition for simplicity) versus when immersed in water.

**Figure 1:**
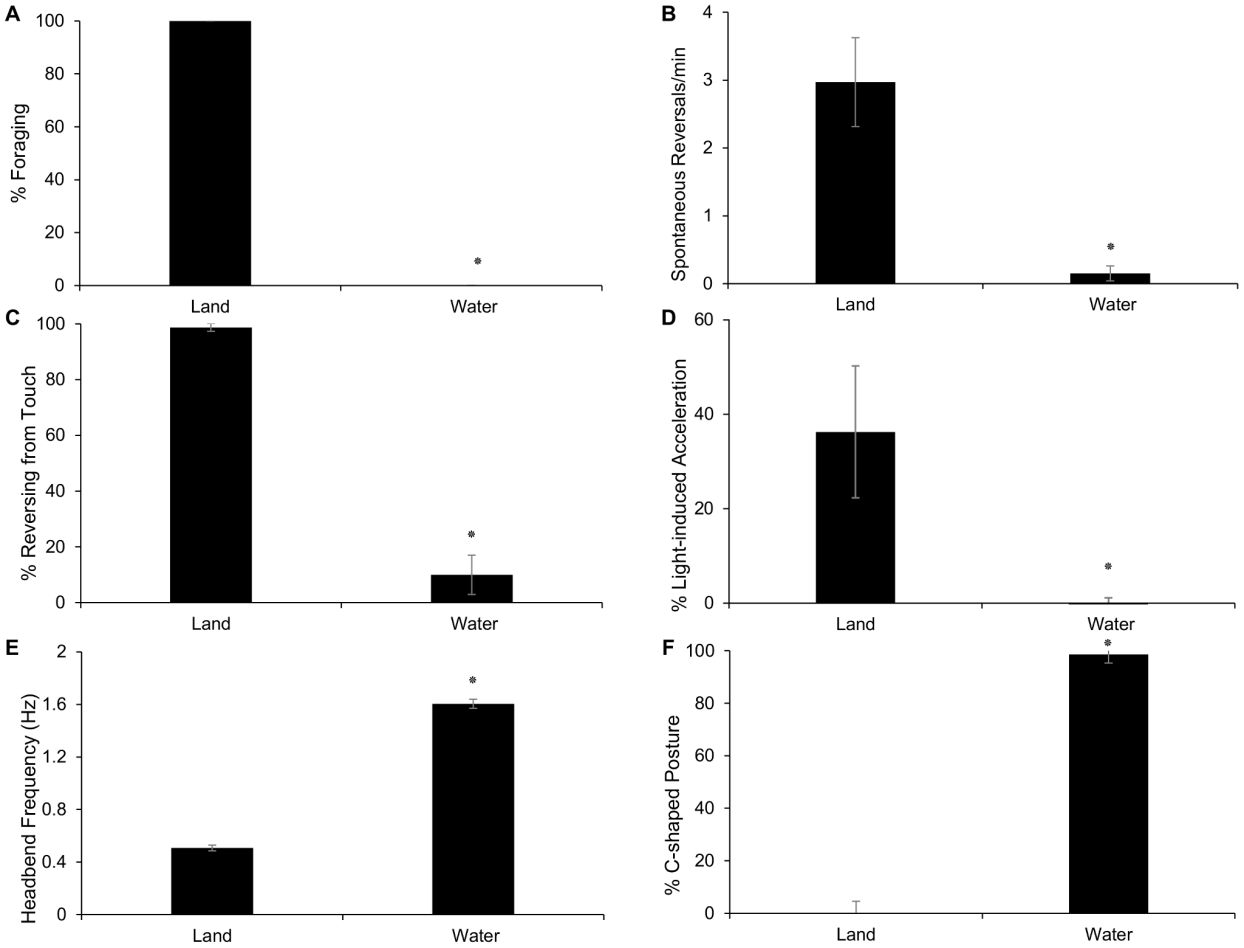
Crawl Behaviors Are Inhibited in Water. Immersion in liquid results in inhibition of many behaviors in wild-type *C. elegans*. Notably, the feeding behavior foraging (A), spontaneous reversals (B), touch response (C), and light response (D) are all inhibited. To assess disinhibition of crawl during immersion in water, headbend frequency (E) and percent body bends with C-shape (F) were assessed. In liquid, worms exhibited only a fast, C-shaped swim. Statistical analyses comparing behaviors on land vs. water were performed using planned unpaired two-tailed t-test. Asterisks indicate P<0.001, n≥4 assays, ≥10 worms per assay for all experiments A–C, n≥15 for D–F. Error bars represent standard error of the mean.

First, we measured the incidence of a behavior called “foraging” that is associated with feeding. Foraging consists of the worm wiggling the anterior-most tip of its head, which contains the sensory organs and mouth, at about 10 Hz [37]. Foraging bends occur in three dimensions and independently from the dorsoventral full-body bends described above for crawling and swimming. Foraging has been proposed to represent a food-seeking behavior, because it occurs most frequently in the presence of food (bacteria) [37]. As in previous reports, we found that worms displayed foraging and pharyngeal pumping on land, but not in water [35], [36] (Figure 1 a).

Second, we tested whether the incidence of locomotor behaviors related to dispersion and escape were distinct on land and in water. Many animals, including humans, rodents, flies, and *C. elegans*, display alternating bouts of extended migration and spontaneous reorienting sharp turns that influence efficiency of local search and rates of dispersion. The primary means of reorienting in *C. elegans* is by temporarily moving backwards for 5–10 seconds in a so-called “reversal”. As in our previous study [36], we found that worms displayed three spontaneous reversals per minute on land, but rarely exhibited reversals in water (Figure 1 b). *C. elegans* will also perform a reversal in response to mechanical stimuli [38], [39]. Animals touched near the midbody with a platinum wire reversed away from the stimulus. We found that on land, this effect was seen in over 90% of animals, while immersion in water reduced this behavior drastically (Figure 1 c). Blue light is another noxious stimulus to worms (∼470 nm wavelength) [40]. Animals exposed to blue light rapidly accelerated away from light, increasing their frequency of bending on land, but not in water (Figure 1 d).

Third, we quantified kinematic aspects of forward locomotion that distinguish the crawling and swimming gaits. During crawling, the worm lies on its left or right side while bending its head dorsoventrally at ∼0.5 Hz (Figure 1 e). These bends propagate backwards along the body, causing the worm to form a traveling S-shaped posture during crawling (Figure 1 f). By contrast, during swimming, the worm bends its head dorsoventrally at ∼1.6 Hz (Figure 1 e). Swimming is also distinguished from crawling by bends that are synchronized to form a C-shaped body posture twice per locomotor cycle – a posture that is never displayed on land during crawling (Figure 1 f).

### Ethanol induces disinhibition of specific behaviors in *C. elegans*

After quantitatively characterizing the inhibition of different worm behaviors by immersion in water, we next examined whether EtOH disinhibited any of these behaviors. We compared the responses of wild-type worms immersed in liquid to those immersed in EtOH. Previous work has shown that *C. elegans* exposed to an exogenous concentration of 500-mM EtOH on land displays a gradual decline in locomotion, feeding, and egg-laying behaviors, and eventually becomes immobile over 30 minutes [31]. Intoxication in liquid at the same concentration was found to result in a steady decrease in locomotion over 6 minutes, after which locomotor rate remained constant [41]. While 500-mM EtOH is well above physiologically relevant levels, Alaimo *et al* (2012) demonstrated that this high exogenous dose resulted in an internal EtOH concentration relevant to human consumption and disinhibition in rodents models [1], [4]–[7], [18], [30], [32], [41].

**Figure 2:**
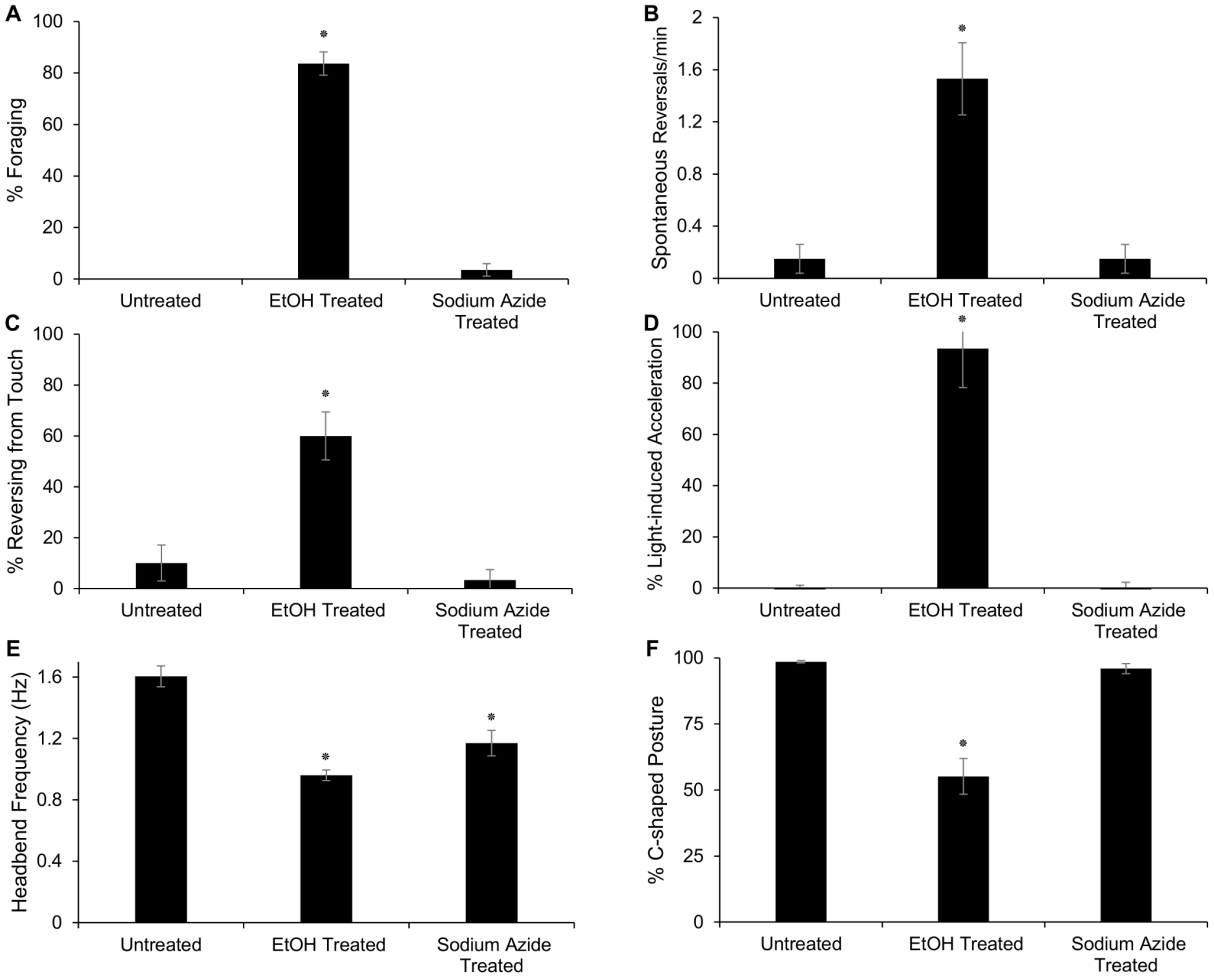
Ethanol Exposure during Immersion in Liquid Results in Disinhibition of Crawl Behaviors. Foraging (A), spontaneous reversals (B), touch response (C), and light response (D), as well as crawling kinematics (E,F) were disinhibited by EtOH. To ensure that such disinhibition was not the result of a decline in cellular function, worms treated with sodium azide were also assessed. No disinhibition was observed in these animals. EtOH treatment resulted in a reduction of bending frequency and a loss of C-shaped body posture. Animals treated with sodium azide experienced a similar decline in bending frequency, but no reduction in C-shape body posture. Statistical analyses comparing EtOH-, azide-, and untreated worms were performed using one-way ANOVA and Tukey's HSD post-hoc test or Kruskal-Wallis and Steel-Dwass-Critchlow-Fligner post-hoc test. Asterisks indicate significance in relation to untreated controls with P<0.001, n≥4 assays, ≥10 worms per assay for all experiments A–C, n≥15 for D–F. Error bars represent standard error of the mean.

We found that animals exposed to EtOH during immersion in liquid displayed disinhibition of several behaviors that are never (or rarely) observed in water. These included foraging, spontaneous reversal, touch response, and blue light response (Figure 2 a–d). To test whether this effect of EtOH on worms in water was distinct from a generic decline in locomotion performance, animals were treated with 1-mM sodium azide. This compound inhibits cellular respiration, resulting in a gradual decline of cellular activity [42], [43]. Animals treated with sodium azide displayed locomotor decline soon after application. After 7 minutes, head-bend frequencies of sodium azide-treated worms were similar to those of EtOH-treated animals (Figure 2 e). However, despite their lower locomotion rate, these animals did not display significant disinhibition of spontaneous reversals, foraging, touch response, or light response (Figure 2 a–d). In addition, these animals displayed mostly C-shaped body postures characteristic of swim, while animals exposed to EtOH showed significantly fewer C-shaped postures characteristic of swimming (Figure 2 f) and more crawl-like S-shaped postures.

### Disinhibition of Foraging is Partially Dependent on D1-like Dopamine Signaling

**Figure 3:**
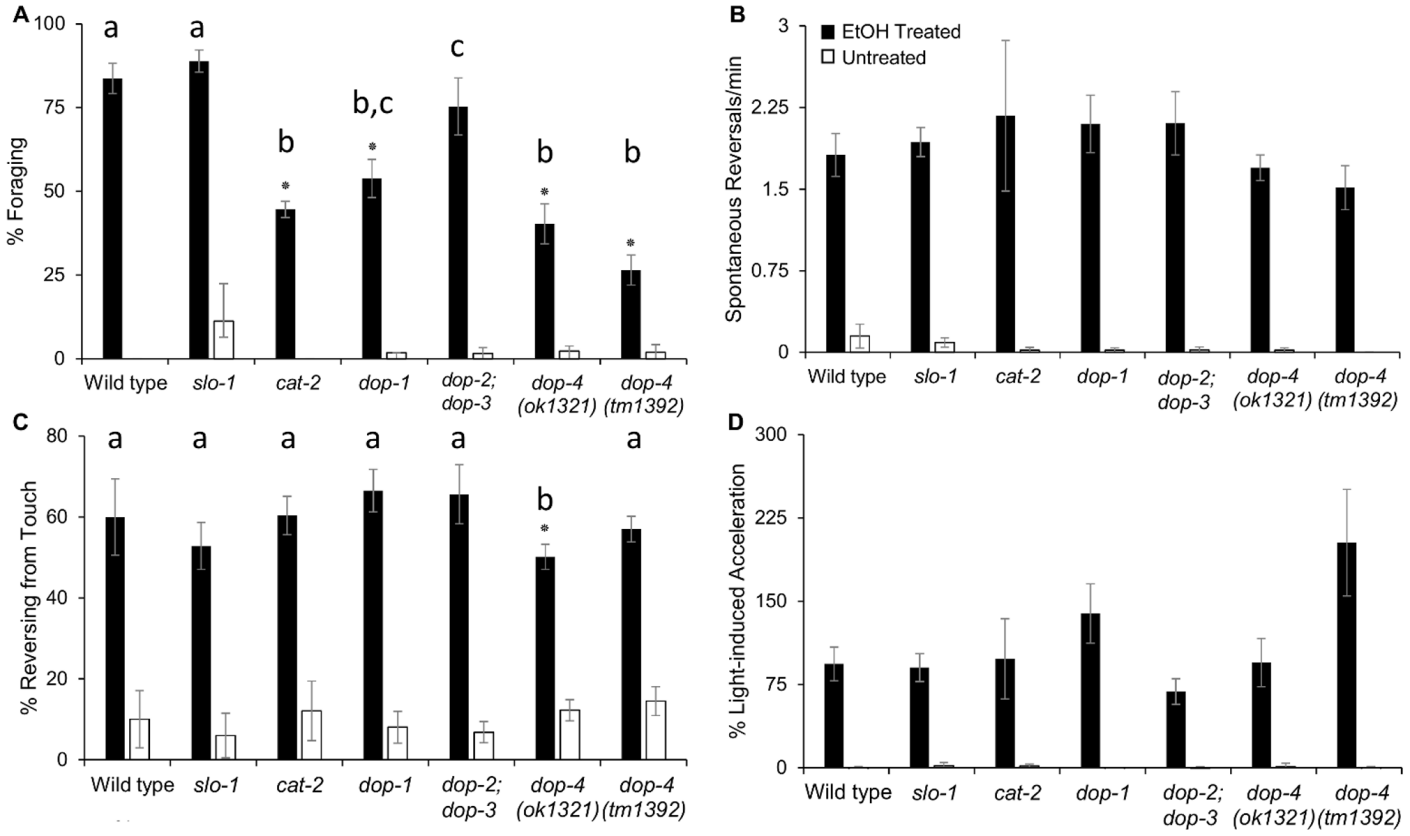
Disinhibition of Foraging Involves Dopamine Signaling. Loss of dopamine synthesis or D1-like dopamine signaling significantly reduced disinhibition of foraging (A). No significant reduction in disinhibition of spontaneous reversals or touch response was observed in animals lacking SLO-1, dopamine synthesis, or dopamine receptors (B,C) except for *dop-4(ok1321)*. Although slightly varied, responses to light did not differ significantly among strains as determined by post-hoc analyses (D). Statistical analyses comparing EtOH-treated mutants to EtOH-treated WT controls were performed using one-way ANOVA and Tukey's HSD post-hoc test or Kruskal-Wallis and Steel-Dwass-Critchlow-Fligner post-hoc test. Letters indicate distinct groupings based on post-hoc statistical comparison among strains. Asterisks indicate significance in relation to WT controls (EtOH-treated or untreated, accordingly) with P<0.001, n≥4 assays, ≥10 worms per assay for all experiments. Error bars represent standard error of the mean.

Our previous work has shown that transition from swimming to crawling is initiated by dopamine release and D1-like dopamine receptor signaling [35]. To investigate the role of the dopaminergic system in the EtOH-induced disinhibition of behaviors, animals deficient in dopamine signaling were evaluated [44]–[47]. We found that disruption of dopamine synthesis by deletion of the worm tyrosine hydroxylase gene, *cat-2*, partly, but significantly, reduced EtOH-induced disinhibition of foraging behavior (Figure 3 a). Likewise, deletion of D1-like dopamine receptor genes *dop-1* or *dop-4* also reduced disinhibition of foraging (Figure 3 a). We observed the same result for two deletion alleles of *dop-4*, raising the likelihood that this phenotype corresponded with loss of function of the *dop-4* gene. By contrast, deletion of the D2-like receptor genes *dop-2* and *dop-3* in combination had no effect versus WT on disinhibition of foraging (Figure 3 a). Post-hoc statistical analysis of this strain revealed minor differences from WT and similarity to the *dop-1* mutant. As dopamine has been previously shown to activate foraging in water [35], this may point to a partial role for D1-like dopamine signaling in the response to EtOH. Likewise, just as in our previous study [35], more salient effects were found for loss of the DOP-4 receptor versus loss of DOP-1 receptor.

Intriguingly, disruption of dopamine signaling via mutation generally did not alter the level of EtOH-induced disinhibition for other crawl-associated behaviors. This included spontaneous reversals, touch-induced reversals, and acceleration in response to blue light (Figure 3 b–d). Post-hoc statistical analysis revealed a slightly lower response to touch for the *dop-4* mutant allele *ok1321* (Figure 3c); however, the other *dop-4* allele *tm1392* did not share this phenotype, suggesting it may not be attributed to loss of the *dop-4* gene. Taken together, these data suggest that a pathway other than dopamine influences these additional aspects of EtOH-induced disinhibition.

One possible pathway for EtOH-induced disinhibition is via the BK potassium channel. Two previous genetic screens revealed that the BK channel SLO-1 was a direct target of EtOH and the major modulator of acute depressive responses to EtOH for crawling and egg laying behaviors in *C. elegans* [32]. This channel is widely expressed in the neurons and muscles, and loss of SLO-1 enhances neurotransmitter release [48]. Thus, it is possible that EtOH-induced disinhibition acts through SLO-1, and its loss would decrease the observed EtOH sensitivity. We found, however, that *slo-1* mutant animals displayed a wild-type level of disinhibition for all quantified behaviors (Figure 3 a–d; raw data for % light-induced acceleration found in Table S1). This strongly indicates that disinhibition is not the result of generalized action of EtOH across the nervous system via this central target of intoxication.

### Disinhibition of crawling is dependent on the D1-like dopamine receptor DOP-4

**Figure 4:**
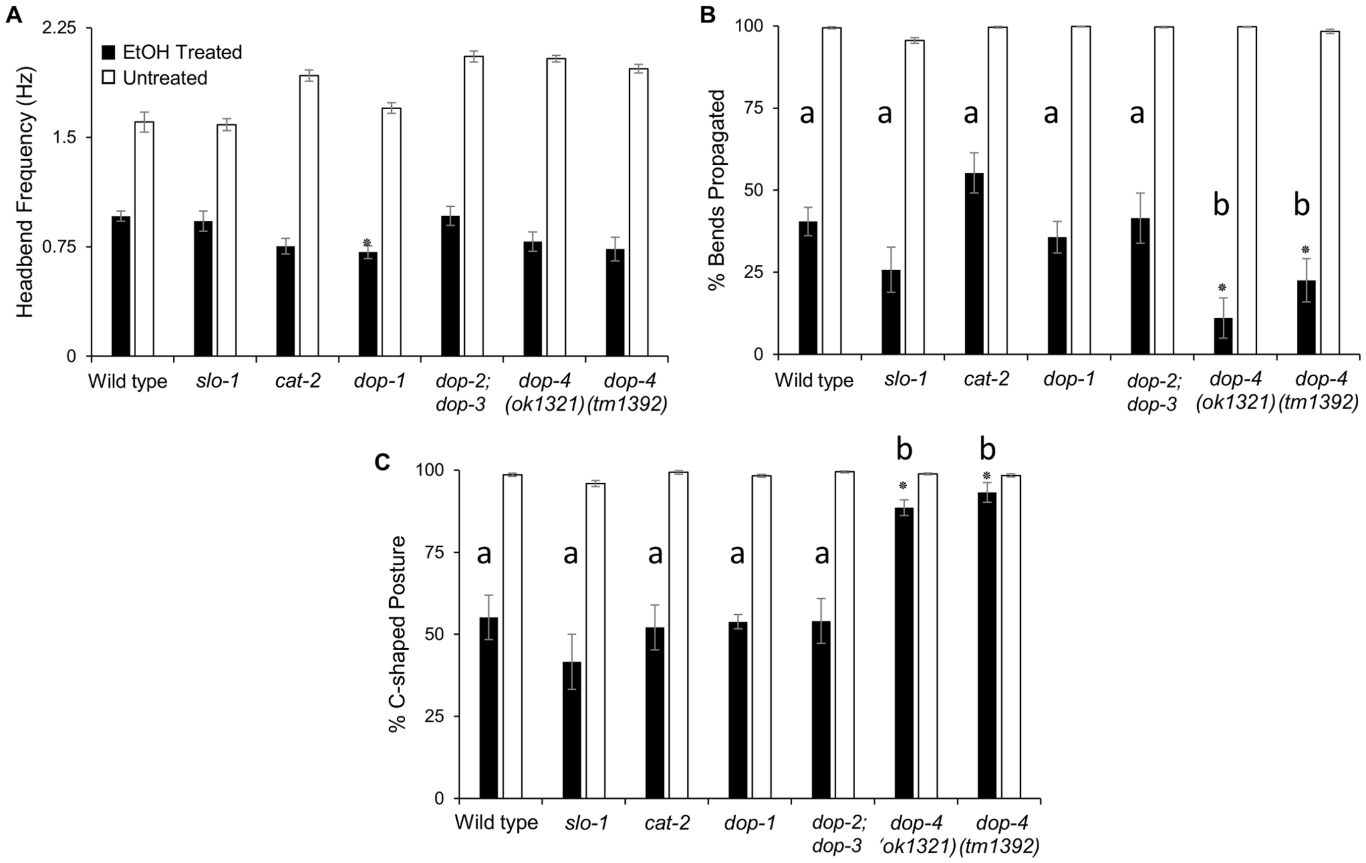
Loss of D1-like Dopamine Receptor DOP-4 Reduces Disinhibition of Crawl. Loss of the D1-like receptor DOP-1 resulted in a slightly lower bending frequency versus WT with EtOH treatment (A). EtOH treatment also caused uncoordination, with significantly fewer bends propagated down the animal. This phenotype was exacerbated in *dop-4* mutant animals (B). Of body bends propagated down the animal, approximately half were C-shaped in most intoxicated animals, indicating disinhibition of crawl. Only animals lacking *dop-4* demonstrated resistance to this effect. Statistical analyses comparing EtOH-treated mutants to EtOH-treated WT controls were performed using one-way ANOVA and Tukey's HSD post-hoc test or Kruskal-Wallis and Steel-Dwass-Critchlow-Fligner post-hoc test. Asterisks indicate significance in relation to WT controls (EtOH-treated or untreated, accordingly) with P<0.001, n≥10 worms for all experiments. Letters indicate distinct groupings based on post-hoc statistical comparison among strains. Error bars represent standard error of the mean.

We also investigated whether disinhibition of the crawling locomotor gait depended on dopamine and/or BK channel pathways. When immersed in EtOH, animals lacking the DOP-1 receptor exhibited a slightly lower head bending frequency than wild-type animals (Figure 4 a). In addition, we noticed that many EtOH-treated animals only propagated bends partially down the body or would abnormally move their anterior and posterior halves asynchronously. To quantitatively characterize this uncoordinated motion, the percent of bends that fully propagated along the animal body was calculated. This revealed that the majority of head bends were not propagated during EtOH exposure, even in mutant animals lacking SLO-1 (Figure 4 b). This effect was most prominent in mutant strains lacking *dop-4*, which both propagated significantly fewer bends than wild type (Figure 4 b). The phenotype is most likely due to mutation of the *dop-4* gene because an identical phenotype was found in independent alleles of *dop-4* (Figure 4 b). We previously observed a similar failure of the *dop-4* mutant to propagate bends when attempting to transition to crawling following swimming [35]. Thus, the significantly reduced bending observed in *dop-4* mutants may be due to an inability of these animals to transition from swimming to EtOH-induced crawling.

When considering the subset of locomotor cycles with bends that fully propagated down the body, we noticed that only animals lacking *dop-4* displayed significantly more C-shaped posture (Figure 4 c). These data, along with the lower bending propagation seen in *dop-4* mutant animals suggest that DOP-4 is involved in the disinhibition of crawling gait during intoxication. We conclude that *dop-4* mutant animals are unable to engage in EtOH-induced crawling, and thus become either more uncoordinated or display slower C-shaped swim bends when exposed to EtOH. Interestingly, *cat-2* mutant animals, which lack dopamine did not show a similar reduction in crawl disinhibition. Thus, EtOH in worms may act more directly through DOP-4 itself or a downstream pathway for this aspect of EtOH-induced disinhibition.

## Discussion

Acute exposure to EtOH is known to disinhibit many behaviors. In humans, this includes social, sexual, and locomotor behaviors [1]–[5], [8], [9]. Such disinhibition has also been demonstrated in fly and rodent models [10]–[12], [18], [30]. This disinhibition was shown to be reliant on the D1 class of dopamine receptors in flies and rodents. Our present study demonstrated a similar effect in *C. elegans*. First, we have developed a novel paradigm to study EtOH-induced disinhibition of behavior in *C. elegans*. Second, we have shown that some disinhibitory effects are dependent in part on dopamine signaling. Third, we found evidence that EtOH may act directly on a D1-like dopamine receptor or downstream pathway. Together, these findings provide an excellent model to study disinhibition and provide evidence for a role of dopamine in the response to EtOH in *C. elegans*.

### *C. elegans* as a model for EtOH-induced disinhibition of behavior

*C. elegans* has previously been shown to display many behavioral effects of EtOH seen in other model animals. It was previously described that *C. elegans* displays acute intoxication, acute tolerance, EtOH preference, and withdrawal [31]–[34], [49]. Our study now adds an important fifth EtOH-induced behavior to this list: disinhibition. This worm model offers many benefits to traditional models of disinhibition, as *C. elegans* quickly matures to genetically identical adults, offers fast generation of transgenic animals, and has a completely described nervous system. In addition, the effects of EtOH on *C. elegans* are robust and easily quantifiable.

Previous studies have shown that several behaviors, including foraging, spontaneous reversal, and crawl are inhibited in water. We further demonstrate that escape responses to blue light and touch are also inhibited in liquid. Upon exposure to EtOH while immersed in liquid, all of these behaviors are disinhibited. This disinhibition was not a result of generalized locomotor or behavioral decline, as disinhibition was not observed in the animals treated with sodium azide. A straight-forward inhibition of swimming would be expected to cause a non-specific decline in locomotor patterns. Instead, we observed that EtOH induced bouts of crawling and a subset of crawl-associated behaviors (e.g. foraging and reversals) that all require coordinated motion. From these results, we conclude that EtOH should be viewed as specifically disinhibiting crawl behaviors rather than inhibiting swimming.

### Dopamine is required for disinhibition of foraging in *C. elegans*

Dopamine has been shown to be a key component of acute EtOH intoxication. In mammals, a large body of evidence has demonstrated that dopamine and D1-like dopamine receptors play an important role in EtOH-induced disinhibition of locomotion. The increase in dopamine release following EtOH intoxication is correlated with locomotor disinhibition in rodents [26]. Several studies have shown a sensitization to the disinhibitory effects of EtOH following pretreatment with dopamine reuptake inhibitors or D1 receptor agonists, though this effect is not consistent amongst all rodent models [27]–[29]. However, recent work in *Drosophila* has also demonstrated a role for dopamine and the D1 dopamine receptors in EtOH-induced disinhibition. Loss of dopamine signaling reduced EtOH disinhibition of male-male courtship [18], while loss of D1 dopamine receptors reduced EtOH disinhibition of locomotion [30].

Previously, the only known interaction between dopamine and EtOH in *C. elegans* was the requirement for dopamine in EtOH preference [50]. We found that EtOH showed potent disinhibition of crawling, spontaneous reversals, and touch and light response in worms immersed in liquid. Disinhibition was not modulated by the SLO-1 potassium channel, the major target of EtOH in *C. elegans* [31], indicating disinhibition is distinct from SLO-1-mediated acute intoxication and is instead mediated by other targets. Interestingly, dopamine signaling did not appear to play a role in disinhibition of spontaneous reversals or response to touch and light. Thus, these behaviors may not be induced by the same dopamine signal as the transition to crawl. As EtOH affects a wide variety of targets, including nicotinic and glutamate receptors, this result is not surprising [50], [51]. In addition, a major neuron responsible for harsh touch transduction, PVD, expresses both such receptor subtypes [52]–[55]. We found that dopamine signaling is important in the induction of foraging in immersed *C. elegans*. Previously, it was shown that both dopamine and D1-like receptors are required for initiation of crawling [35], and foraging can be induced in animals immersed in liquid through application of dopamine [36]. Complementing this result, we found that animals lacking dopamine synthesis or D1-like dopamine receptors display significantly less disinhibition of foraging. This points toward a potentially conserved mechanism for disinhibition in *C. elegans* and higher animals.

### Ethanol may act directly on a D1-like dopamine receptor pathway

Unexpectedly, we found evidence that EtOH may act directly on the D1-like dopamine receptor DOP-4 or through its downstream signaling. Disinhibition of crawling, as assessed by presence of C-shaped posture, was only seen in animals lacking DOP-4 and not in animals lacking dopamine or SLO-1. Previous research in mice found extensive evidence for a role of D1-like dopamine receptors in locomotor disinhibition via EtOH. While there are many links between D1-like dopamine receptors and EtOH-induced disinhibition, these have been attributed to the increase in dopamine observed following acute intoxication. Many papers have pointed towards a role for D1-like receptors in the disinhibition of locomotion and EtOH-seeking behaviors [23]–[25], [27]–[29]. We are not aware of any papers demonstrating any direct interaction of EtOH on dopamine receptors. Thus, this work demonstrates a potential novel effect of EtOH on D1-like receptors that is independent of dopamine release.

## Experimental Procedures

### Animals

*C. elegans* were grown on nematode growth media (NGM) agar plates seeded with OP50 bacteria at 20°C as previously described [56]. Mutant strains were obtained from the Caenorhabditis Genetic Center and the *C. elegans* Gene Knockout Consortium. The following strains were used: WT N2, *cat-2(e112)*II, *dop-1(vs101)*X, *dop-1(vs100)*X, *dop-4(ok1321)*X, *dop-4(tm1392)*X, *dop-2(vs105)V;dop-3(vs106)*X, and *slo-1(js118)*V.

### Pharmacological Assays

Each EtOH assay was conducted on 10–15, never-starved, young adult worms. Worms were cleaned of bacteria by allowing them to crawl on an empty plate for 2 minutes before each experiment. Assays were performed on plates containing 500-mM EtOH in the agar medium. 10–15 animals were picked into a 6-μL drop of 500-mM EtOH (200 proof; Sigma-Aldrich, St. Louis MO). EtOH solution was prepared by adding 200-proof EtOH to standard nematode growth medium (NGM). As osmolarity is known to affect intoxication, NGM was tested prior to experiments to ensure a constant 180 mOsm. Worm behavior was recorded for 30 minutes continuously. Additional 6-μL drops of 500-mM EtOH were added every 2–3 minutes, when the boundary of the drop began to recede. Worms maintain swimming and suppress crawling behaviors as long as the depth of the drop is greater than the width of the worm [35]. Control assays were performed in the same manner, except EtOH was not added to NGM media. Previously reported internal EtOH concentrations after 10 minutes of 500-mM EtOH exposure ranged from 17.5–67.5 mM for animals on land [41]. This correlates well to disinhibiting doses seen in rodent and human disinhibition studies [4]–[7], [18], [30]. Internal EtOH concentration may be lower than those previously reported given that immersion in water inhibits ingestion by pharyngeal pumping [35]. Movie recordings were made at 30 frames/s, 344 pixels/mm using a Flea2 camera (Point Grey Research, Richmond, Canada) and StreamPix software (NorPix, Montreal, Canada). Sodium azide assays were performed by placing a 6-μL drop of 1-mM sodium azide (Sigma-Aldrich) onto a thin pad of agarose. 10–12 worms were then placed inside the drop and their activity was recorded for 30 minutes. Additional 6-μL drops of 1-mM sodium azide were added as needed.

To quantify different behaviors, groups of animals were analyzed for a 1-minute time window after 7 minutes of EtOH exposure at the beginning of the 30-minute recording. Foraging: Foraging was assessed by presence of ∼5–10 Hz bending of the tip of the nose for each worm. Percent animals foraging was quantified by number of animals in a group displaying foraging behavior over one minute divided by total number of animals. Bending frequency: Head-bend duration was defined as the time the head traveled from its maximal dorsal flexure to maximal ventral flexure and vice versa. Head bends that did not change from ventral to dorsal flexure (or vice versa) were not counted, nor were bends that did not propagate down the body. Bend Propagation: Bending propagation was quantified by dividing number of bends initiated at the head of an animal divided by bends propagated to the tail. Posture: To characterize body posture, at the apex of each bend a line was drawn from nose to tail. If this line did not intersect the body at any point, then the animal was considered C-shaped. Only bends propagated down the body were analyzed for posture. Reversals: Reversals were defined as a backward movement spanning a distance greater than the pharynx of the animal. Touch Response: Touch response assays were also performed after 7 minutes of intoxication in a 6-μL drop of EtOH. The head of each animal was gently prodded with a platinum wire and a touch response was considered positive if the animal initiated a reversal after prodding. Light Response: To assay blue light response, animal behavior was recorded for 1 minute. The animal was then exposed to 1.6 mW/mm^2^ 420-nM wavelength blue light from a Prior Lumen200 fluorescent light system for 30 seconds. Head-bending frequencies were counted before and after illumination and the percent increase for each animal was determined.

### Statistical Analyses

Statistical analyses were performed using JMP 11.0.0 SAS Software (SAS Institute, Cary, NC, USA). Results are presented as the mean ± standard error. Crawl behavior inhibition in water (Figure 1) was tested by unpaired two-tailed t-test. The statistical significance of differences between EtOH-, azide-treated, and untreated worms (Figure 2), ethanol-treated mutant populations vs. ethanol-treated wild-type worms, and untreated mutant populations vs. untreated wild-type worms (Figures 3 and 4) was demonstrated by a one-way analysis of variance (ANOVA), with Tukey's honest significant difference (HSD) multiple comparison post-hoc tests. If the data did not meet the assumptions for ANOVA test, data were analyzed using a Kruskal-Wallis test followed by a Steel-Dwass-Critchlow-Fligner multiple comparison test between each group. All differences were considered significant at P<0.001 (see Figures 1, 2, 3, 4).

## Supporting Information

Table S1 Raw data broken into groups of genotype and treatment used to calculate percentage acceleration in response to blue light for Figure 3D. Paired values represent number of head bends observed in two 60 second time windows before and after blue light for different individual worms. Each worm was recorded and exposed to blue light only once.
(PDF)

## References

[1] Moberg CA, Curtin JJ (2009) Alcohol selectively reduces anxiety but not fear: startle response during unpredictable versus predictable threat. J Abnorm Psychol 118: 335–47. 19413408 10.1037/a0015636 PMC2756160

[2] de Boer MC, Schippers GM, van der Staak CP (1993) Alcohol and social anxiety in women and men: pharmacological and expectancy effects. Addict Behav 18: 117–26. 8506782 10.1016/0306-4603(93)90041-7

[3] Rose AK, Duka T (2007) The influence of alcohol on basic motoric and cognitive disinhibition. Alcohol Alcohol 42: 544–51. 17878213 10.1093/alcalc/agm073

[4] Weafer J, Fillmore MT (2012) Comparison of alcohol impairment of behavioral and attentional inhibition. Drug Alcohol Depend 126: 176–82. 22673197 10.1016/j.drugalcdep.2012.05.010 PMC3440541

[5] Marinkovic K, Halgren E, Klopp J, Maltzman I (2000) Alcohol effects on movement-related potentials: a measure of impulsivity? J Stud Alcohol 61: 24–31. 10627093 10.15288/jsa.2000.61.24

[6] Babor TF, Berglas S, Mendelson JH, Ellingboe J, Miller K (1982) Alcohol, affect, and the disinhibition of verbal behavior. Psychopharmacology Berl 80: 53–60. 10.1007/BF00427496 6408672

[7] Sayette MA, Creswell KG, Dimoff JD, Fairbairn CE, Cohn JF, et al (2012) Alcohol and group formation: a multimodal investigation of the effects of alcohol on emotion and social bonding. Psychol Sci 23: 869–78. 22760882 10.1177/0956797611435134 PMC5462438

[8] Prause N, Staley C, Finn P (2011) The Effects of Acute Ethanol Consumption on Sexual Response and Sexual Risk-Taking Intent. Archives of Sexual Behavior 40: 373–384. 21318417 10.1007/s10508-010-9718-9

[9] Stoner S, George WH, Peters LM, Norris J (2007) Liquid Courage: Alcohol Fosters Risky Sexual Decision-Making in Individuals with Sexual Fears. AIDS and Behavior 11: 227–237. 16802196 10.1007/s10461-006-9137-z

[10] Ahlenius S, Brown R, Engel J, Svensson TH, Waldeck B (1974) Short communication. Antagonism by nialamide of the ethanol-induced locomotor stimulation in mice. J Neural Transm 35: 175–8. 4844105 10.1007/BF01250743

[11] Imperato A, Di Chiara G (1986) Preferential stimulation of dopamine release in the nucleus accumbens of freely moving rats by ethanol. J Pharmacol Exp Ther 239: 219–28. 3761194

[12] Varlinskaya EI, Spear LP (2012) Increases in anxiety-like behavior induced by acute stress are reversed by ethanol in adolescent but not adult rats Pharmacol Biochem Behav. 100: 440–450. 10.1016/j.pbb.2011.10.010 PMC3256739 22024161

[13] Pisu MG, Mostallino MC, Dore R, Maciocco E, Secci PP, et al (2011) Effects of voluntary ethanol consumption on emotional state and stress responsiveness in socially isolated rats. Eur Neuropsychopharmacol 21: 414–425. 21067904 10.1016/j.euroneuro.2010.07.006 PMC3044778

[14] Pohorecky LA (2008) Psychosocial stress and chronic ethanol ingestion in male rats: effects on elevated plus maze behavior and ultrasonic vocalizations. Physiol Behav 94: 432–447. 18417174 10.1016/j.physbeh.2008.02.010

[15] Gomez JL, Lewis MJ, Luine VN (2012) The interaction of chronic restraint stress and voluntary alcohol intake: effects on spatial memory in male rats. Alcohol 46: 499–504. 22560292 10.1016/j.alcohol.2011.12.005 PMC3389186

[16] Colombo G, Agabio R, Lobina C, Reali R, Zocchi A, et al (1995) Sardinian alcohol-preferring rats: a genetic animal model of anxiety. Physiol Behav 57: 1181–1185. 7652041 10.1016/0031-9384(94)00382-f

[17] Pandey SC, Zhang H, Roy A, Xu TJ (2005) Deficits in amygdaloid cAMP-responsive element-binding protein signaling play a role in genetic predisposition to anxiety and alcoholism. Clin Invest 115: 2762–2773. 10.1172/JCI24381 PMC1236671 16200210

[18] Lee HG, Kim YC, Dunning JS, Han KA (2008) Recurring ethanol exposure induces disinhibited courtship in Drosophila. PLoS ONE 3: e1391. 18167550 10.1371/journal.pone.0001391 PMC2148075

[19] Weiss F, Lorang MT, Bloom FE, Koob GF (1993) Oral alcohol self-administration stimulates dopamine release in the rat nucleus accumbens: genetic and motivational determinants. J Pharmacol Exp Ther 267: 250–258. 8229752

[20] Imperato A, Di Chiara G (1986) Preferential stimulation of dopamine release in the nucleus accumbens of freely moving rats by ethanol. J Pharmacol Exp Ther 239: 219–228. 3761194

[21] Yim HJ, Gonzales RA (2000) Ethanol-induced increases in dopamine extracellular concentration in rat nucleus accumbens are accounted for by increased release and not uptake inhibition. Alcohol 22: 107–115. 11113625 10.1016/s0741-8329(00)00121-x

[22] Gonzales RA, Job MO, Doyon WM (2004) The role of mesolimbic dopamine in the development and maintenance of ethanol reinforcement. Pharmacol Ther 103: 121–146. 15369680 10.1016/j.pharmthera.2004.06.002

[23] Hodge CW, Samson HH, Chappelle AM (1997) Alcohol self administration: further examination of the role of dopamine receptors in the nucleus accumbens. Alcohol Clin Exp Res 21: 1083–1091. 9309321 10.1111/j.1530-0277.1997.tb04257.x PMC11584055

[24] Rassnick S, Pulvirenti L, Koob GF (1992) Oral ethanol self administration in rats is reduced by the administration of dopamine and glutamate receptor antagonists into the nucleus accumbens. Psychopharmacol 109: 92–98. 10.1007/BF02245485 1365677

[25] Samson HH, Hodge CW, Tolliver GA, Haraguchi M (1993) Effect of dopamine agonists and antagonists on ethanol-reinforced behavior: the involvement of the nucleus accumbens. Brain Res Bull 30: 133–141. 8093596 10.1016/0361-9230(93)90049-h

[26] Melendez RI, Rodd-Henricks ZA, Engleman EA, Li TK, McBride WJ, et al (2002) Microdialysis of dopamine in the nucleus accumbens of alcohol-preferring (P) rats during anticipation and operant self-administration of ethanol. Alcohol Clin Exp Res 26: 318–325. 11923583

[27] Abrahao KP, Quadros IM, Souza-Formigoni ML (2011) Nucleus accumbens dopamine D_1_ receptors regulate the expression of ethanol-induced behavioural sensitization. Int J Neuropsychopharmacol 14: 175–185. 20426882 10.1017/S1461145710000441

[28] Broadbent J, Kampmueller K, Koonse SA (2005) Role of dopamine in behavioral sensitization to ethanol in DBA/2J mice. Alcohol 45: 137–148. 10.1016/j.alcohol.2005.03.006 15963427

[29] Bahi A, Dreyer JL (2012) Involvement of tissue plasminogen activator “tPA” in ethanol-induced locomotor sensitization and conditioned-place preference. Behav Brain Res 226: 250–258. 21945298 10.1016/j.bbr.2011.09.024

[30] Kong EC, Woo K, Li H, Lebestky T, Mayer N, et al (2010) A pair of dopamine neurons target the D1-like dopamine receptor DopR in the central complex to promote ethanol-stimulated locomotion in Drosophila. PLoS ONE 5: e9954. 20376353 10.1371/journal.pone.0009954 PMC2848596

[31] Davies AG, Pierce-Shimomura JT, Kim H, VanHoven MK, Thiele TR, et al (2003) A central role of the BK potassium channel in behavioral responses to ethanol in *C. elegans* . Cell 115: 655–666. 14675531 10.1016/s0092-8674(03)00979-6

[32] Mitchell PH, Bull K, Glautier S, Hopper NA, Holden-Dye L, et al (2007) The concentration-dependent effects of ethanol on *Caenorhabditis elegans* behaviour. J Pharmacogenomics 7: 411–417. 10.1038/sj.tpj.6500440 17325734

[33] Davies AG, Bettinger JC, Thiele TR, Judy ME, McIntire SL (2004) Natural variation in the *npr-1* gene modifies ethanol responses of wild strains of *C. elegans* . Neuron 42: 731–743. 15182714 10.1016/j.neuron.2004.05.004

[34] Mitchell P, Mould R, Dillon J, Glautier S, Andrianakis I, et al (2010) A differential role for neuropeptides in acute and chronic adaptive responses to alcohol: behavioural and genetic analysis in *Caenorhabditis elegans* . PLoS ONE 5: e10422. 20454655 10.1371/journal.pone.0010422 PMC2862703

[35] Vidal-Gadea A, Topper S, Young L, Crisp A, Kressin L, et al (2011) *Caenorhabditis elegans* selects distinct crawling and swimming gaits via dopamine and serotonin. Proc Natl Acad Sci 108: 17504–17509. 21969584 10.1073/pnas.1108673108 PMC3198358

[36] Vidal-Gadea AG, Davis S, Becker L, Pierce-Shimomura JT (2012) Coordination of behavioral hierarchies during environmental transitions in *Caenorhabditis elegans* . Worm 1: 5–11. 23525841 10.4161/worm.19148 PMC3606076

[37] De Bono, Bargmann CI (1998) Natural variation in a neuropeptide Y receptor homolog modifies social behavior and food response in *C. elegans* . Cell 94: 679–689. 9741632 10.1016/s0092-8674(00)81609-8

[38] Way JC, Chalfie M (1989) The *mec-3* gene of *Caenorhabditis elegans* requires its own product for maintained expression and is expressed in three neuronal cell types. Genes Dev 3: 1823–1833. 2576011 10.1101/gad.3.12a.1823

[39] Sulston J, Dew M, Brenner S (1975) Dopaminergic neurons in the nematode *Caenorhabditis elegans* . J Comp Neurol 163: 215–226. 240872 10.1002/cne.901630207

[40] Edwards SL, Charlie NK, Milfort MC, Brown BS, Gravlin CN, et al (2008) A novel molecular solution for ultraviolet light detection in *Caenorhabditis elegans* . PLoS Biol 6: e198. 18687026 10.1371/journal.pbio.0060198 PMC2494560

[41] Alaimo JT, Davis SJ, Song SS, Burnette CR, Grotewiel M, et al (2012) Ethanol metabolism and osmolarity modify behavioral responses to ethanol in *C. elegans* . Alcohol Clin Exp Res 36: 1840–1850. 22486589 10.1111/j.1530-0277.2012.01799.x PMC3396773

[42] Herweijer MA, Berden JA, Kemp A, Slater EC (1985) Inhibition of energy-transducing reactions by 8-nitreno-ATP covalently bound to bovine heart submitochondrial particles: direct interaction between ATPase and redox enzymes. Biochim Biophys Acta 809: 81–89. 2862915 10.1016/0005-2728(85)90170-7

[43] Duncan HM, Mackler B (1966) Electron transport systems of yeast. 3. Preparation and properties of cytochrome oxidase. J Biol Chem 241: 1694–1697. 4287709

[44] Suo S, Sasagawa N, Ishiura S (2002) Identification of a dopamine receptor from *Caenorhabditis elegans* . Neurosci Lett 319: 13–16. 11814642 10.1016/s0304-3940(01)02477-6

[45] Chase DL, Pepper JS, Koelle MR (2004) Mechanism of extrasynaptic dopamine signaling in *Caenorhabditis elegans* . Nat Neurosci 10: 1096–1103. 10.1038/nn1316 15378064

[46] Sugiura M, Fuke S, Suo S, Sasagawa N, Van Tol HH, et al (2012) Characterization of a novel D2-like dopamine receptor with a truncated splice variant and a D1-like dopamine receptor unique to invertebrates from *Caenorhabditis elegans* . J Neurochem 94: 1146–57. 10.1111/j.1471-4159.2005.03268.x 16001968

[47] Suo S, Sasagawa N, Ishiura S (2003) Cloning and characterization of a *Caenorhabditis elegans* D2-like dopamine receptor. J Neurochem 86: 869–878. 12887685 10.1046/j.1471-4159.2003.01896.x

[48] Wang ZW, Saifee O, Nonet ML, Salkoff L (2001) SLO-1 potassium channels control quantal content of neurotransmitter release at the *C. elegans* neuromuscular junction. Neuron 32: 867–881. 11738032 10.1016/s0896-6273(01)00522-0

[49] Yang X, Criswell HE, Breese GR (1999) Action of ethanol on responses to nicotine from cerebellar interneurons and medial septal neurons: relationship to methyllycaconitine inhibition of nicotine responses. Alcohol Clin Exp Res 23: 983–990. 10397282

[50] Lee J, Jee C, McIntire SL (2009) Ethanol preference in *C. elegans* . Genes Brain Behav 8: 578–585. 19614755 10.1111/j.1601-183X.2009.00513.x PMC2880621

[51] Weight FF, Aguayo LG, White G, Lovinger, D M, Peoples, R W (1992) GABA-and glutamate-gated ion channels as molecular sites of alcohol and anesthetic action. Adv Biochem Psychopharmacol 47: 335–347. 1354918

[52] Mongan NP, Jones AK, Smith GR, Sansom MSP, Sattelle DB (2002) Novel α7-like nicotinic acetylcholine receptor subunits in the nematode *Caenorhabditis elegans* . Protein Sci 11: 1162–1171. 11967372 10.1110/ps.3040102 PMC2373549

[53] Yassin L, Gillo B, Kahan T, Halevi S, Eshel M, et al (2001) Characterization of the DEG-3/DES-2 receptor: A nicotinic acetylcholine receptor that mutates to cause neuronal degeneration. Mol Cell Neurosci 17: 589–1599. 11273652 10.1006/mcne.2000.0944

[54] Sprengel R, Aronoff R, Völkner M, Schmitt B, Mosbach R, Kuner T (2001) Glutamate receptor channel signatures. Trends Pharmacol Sci 22: 7–10. 11165660 10.1016/s0165-6147(00)01588-1

[55] Wintle RF, Van Tol HH (2001) Dopamine signaling in *Caenorhabditis elegans*-potential for parkinsonism research. Parkinsonism Relat Disord 7: 177–183. 11331184 10.1016/s1353-8020(00)00055-9

[56] Brenner S (1974) The genetics of *Caenorhabditis elegans* . Genetics 77: 71–94. 4366476 10.1093/genetics/77.1.71 PMC1213120

