# Supplementary material for: Alcohol Disinhibition of Behaviors in C. elegans
Source: PLoS One. 2014 Mar 28;9(3):e92965. doi: 10.1371/journal.pone.0092965 (PMC3969370; doi:10.1371/journal.pone.0092965)
Supplement: Table S1 — Raw data broken into groups of genotype and treatment used to calculate percentage acceleration in response to blue light for Figure 3D. Paired values represent number of head bends observed in two 60 second time windows before and after blue light for different individual worms. Each worm was recorded and exposed to blue light only once. (PDF) [file pone.0092965.s001.pdf]

Genotype	Treatment	Head bends during 60 seconds (before/during blue-light exposure) for different individual worms
wild-type (N2)	Untreated	90/81, 77/68, 90/70,80/80,80/85,90/86,84/87,78/88,98/89,71/78
	EtOH	41/32, 33/53. 41/42 54/34, 18/28, 20/25, 25/35, 39/31, 13/15,24/32, 77/90,35/66, 26/58, 33/73, 56/72, 23/52, 11/18, 13/47,67/84
slo-1	Untreated	99/97, 86/89, 89/81, 99/116, 94/100, 86/79, 92/94, 94/94, 102/115, 111/106
	EtOH	69/85, 62/102, 72/93, 48/104, 58/96, 67/107, 47/89, 53/87, 40/97, 43/93, 25/60, 38/78, 29/87, 43/76, 46/74
cat-2	Untreated	101/99, 101/103, 97/94, 116/109, 98/108, 100/106, 98/102, 103/104, 99/97, 99/104,
	EtOH	35/56, 12/43, 20/11,30/76, 22/43, 31/60,22/22, 14/11, 25/28, 12/28, 12/16, 14/58, 18/66, 15/16, 4/18, 8/34, 5/20, 6/24, 84/112, 94/92, 98/110, 86/102, 76/103, 96/105, 72/88, 73/101, 72/90, 92/105, 76/108, 74/88, 50/83, 70/87, 62/81, 48/88, 56/67, 59/72, 40/85, 12/55, 25/53, 35/63
dop-1	Untreated	94/88, 88/84, 86/84,93/98, 92/94, 92/89, 101/94, 94/89, 94/99, 84/81
	EtOH	50/67,37/63, 45/67, 33/53, 14/45, 54/59, 40/58, 51/12, 29/35, 20/37, 22/47, 19/60, 33/38, 11/37, 14/25, 14/47, 10/15, 20/25, 7/35
dop-2; dop-3	Untreated	91/90, 104/99, 114/115, 104/94, 104/105, 111/112, 115/122, 106/97, 114/116, 94/99
	EtOH	65/82, 72/85, 48/73, 60/65, 85/103, 63/89, 35/42, 55/68, 56/69, 66/65, 49/60, 69/70, 66/78, 49/73, 42/57, 59/72, 45/59, 57/58, 43/84
dop-4 (ok1321)	Untreated	117/112, 114/111, 101/111, 94/105, 117/96, 96/102, 107/117, 104/111, 120/108, 107/107
	EtOH	17/67, 42/68, 54/84, 46/80, 50/76, 44/70, 38/81, 47/86, 35/84 23/79, 52/58, 65/72, 57/77, 60/89, 21/50
dop-4 (tm1392)	Untreated	125/95, 118/103, 117/110, 116/114, 114/135, 128/135, 119/109, 122/112, 114/124, 109/110
	EtOH	90/84, 100/113, 30/85, 63/76, 70/103, 14/69, 69/70, 18/75, 65/91, 13/27, 6/24, 16/24, 21/41, 12/37, 13/20, 37/47, 12/53, 36/53, 45/59, 12/44

Table S1. Raw data broken into groups of genotype and treatment used to calculate percentage acceleration in response to blue light for Figure 3D. Paired values represent number of head bends observed in two 60 second time windows before and after blue light for different individual worms. Each worm was recorded and exposed to blue light only once.
